# An Atypical Behavior of Metastatic Lung Disease in a Young Woman With Osteosarcoma: A Case Report

**DOI:** 10.7759/cureus.21589

**Published:** 2022-01-25

**Authors:** Anaïs Acquisto, Ivan Duran Derijckere, Riccardo De Angelis

**Affiliations:** 1 Radiology, Institut Jules Bordet, Brussels, BEL; 2 Nuclear Medicine, Institut Jules Bordet, Brussels, BEL

**Keywords:** oncology, late-relapse, computed tomography, osteosarcoma, lung metastasis

## Abstract

Osteosarcoma is an aggressive primary malignant bone tumor with frequent local or metastatic recurrence after surgery. Lungs are the most common site of metastasis. Although the majority of relapses will occur within five years of the initial diagnosis of osteosarcoma, lung metastases may appear a long time after initial presentation. This mechanism is known as late-relapse metastasis, in which dormant metastatic cells undergo reactivation and give rise to metastatic outgrowths. We report a case of a 29-year-old woman diagnosed with osteosarcoma of the left distal tibia. Chest CT performed at the initial presentation showed no metastasis. The patient underwent two cycles of neoadjuvant chemotherapy prior to surgery. Routine follow-up chest CT revealed a lung nodule five years later. A sudden increase in the size of the lung nodule was observed two years later. Histopathological analysis of the lung nodule confirmed osteosarcoma lung metastasis. A better understanding of the various types of aspects and atypical behaviors of osteosarcoma metastases could have a significant impact on the prognosis of the patients.

## Introduction

Osteosarcoma (OS) is the most common primary malignant bone tumor with a worldwide incidence rate ranging from two to five cases per million per year worldwide [1]. In the last years, the survival rate of patients with OS has significantly improved with the use of aggressive systemic chemotherapy combined with surgical treatment [2]. Despite the use of multimodality therapy, patients with localized OS could achieve a 60-70% five-year survival rate, and this falls to 10-30% in case of metastatic disease [2]. The lung is the most common site of metastasis, with 77% to 92% of patients experiencing recurrence at this site [3]. Metastases may appear several years after initial presentation, suggesting a mechanism of dormant metastases reactivating [4]. Also, OS may present calcifying pulmonary metastases, which are rare and may lead to misdiagnosis and late treatment. After recurrence, the average survival is of less than one year [2]. We report a case of atypical presentation and features of OS lung metastasis.

## Case presentation

**Figure 1:**
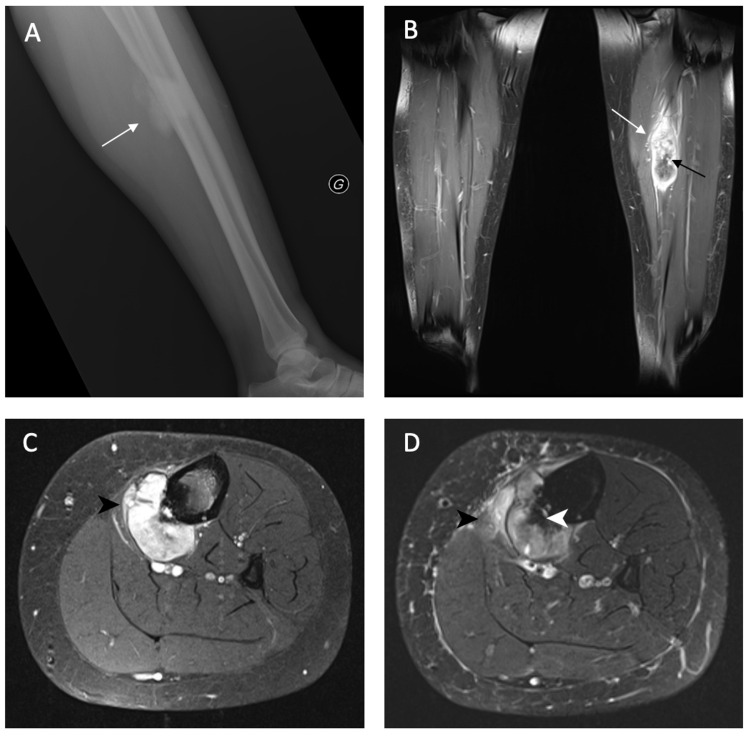
Osteosarcoma of the tibial diaphysis On the initial radiograph (A), the lesion presents as a cortical breakthrough with a dense periosteal bone formation (white arrow) arising from the lateral cortex. The coronal (B) and axial (C) T1-weighted magnetic resonance (MR) images show scattered regions of hemorrhage (black arrow). The axial fat-saturated T2-weighted MR image (D) shows peritumoral soft tissue edema (white arrowhead) and periosteal reaction (black arrowhead).

**Figure 2:**
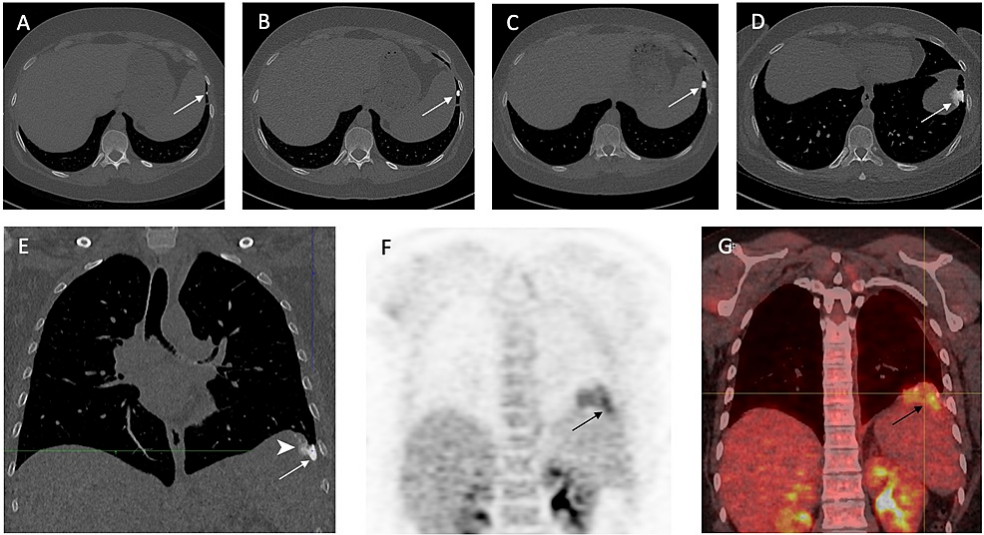
Lung metastasis Initial chest CT scan (A) in 2016 revealed a small calcified lesion in the left lower lobe (white arrow). The lesion remained relatively stable in appearance and size on follow-up chest CT scans in 2018 (B) and 2019 (C), suggestive of a benign process. Axial (D) and coronal (E) chest CT scan in 2021 shows an increase in the size of the lesion with diaphragm invasion (white arrowhead). Coronal 18F-fluorodeoxyglucose positron emission tomography (F) and fused scan (G) images show increased activity of the lesion (black arrow).

In 2014, a 29-year-old woman presented to his primary care physician with a history of chronic pain in his left leg and tiredness. There was a notion of trauma one month earlier. Physical examination and blood tests were overall normal. A plain radiograph was performed and showed a lobulated and exophytic mass with central dense ossification adjacent with periosteal reaction to the bone of the left distal tibia (Figures 1A, 1B). The first suspicion was a neoplastic lesion of the left distal tibia, so the patient was referred to an oncological center where magnetic resonance imaging (MRI) was performed. The MRI showed an exophytic centered on the distal tibia with a periosteal reaction and invasion of adjacent soft tissue (Figures 1C, 1D). Characteristics of this lesion were consistent with the diagnosis of OS. After a multidisciplinary discussion, an ultrasound-guided biopsy was performed. Histolopathogical analysis of the specimen confirmed epithelioid OS. A chest computed tomography (CT) was performed to look for metastatic disease. CT showed no metastasis. The patient underwent two cycles of neoadjuvant chemotherapy (cisplatin and doxorubicin) prior to surgery. Post-surgery histological examination revealed complete resection, with less than 50% necrosis. After surgery, the patient underwent a systematic follow-up, including an MRI of the limb and chest CT (Figures 2A-2C). No signs of recurrence or metastases were observed during the next five years of follow-up. In 2019, a chest CT revealed a unique millimetric nodule, partially calcified, in the posterobasal region of the left lung. During the next two years, this nodule was strictly stable and therefore classified as a sequela. Nonetheless, a CT performed in January 2021 revealed a sudden increase of size from 6 to 22 mm with signs of diaphragmatic invasion (Figures 2D, 2E). An 18F-fluorodeoxyglucose positron emission tomography/CT revealed increased activity of the lung nodule, suggesting malignancy (Figures 2F, 2G). Diagnosis of a reactivation of a late relapse metastasis was proposed. The patient underwent left thoracotomy with wedge resection of the lung nodule. The histological analysis from the resected specimen confirms the diagnosis of lung metastasis from OS. After surgery, the patient underwent adjuvant chemotherapy (Ifosfamide and etoposide). The patient is now followed-up by serial CT scans and is free of metastatic lesions.

## Discussion

OS is the most common primary malignant bone tumor with a worldwide incidence rate ranging from two to five cases per million per year worldwide between 1973 and 2004 [1]. It is also one of the most aggressive kinds of neoplastic lesions, with 30-50% of local or metastatic recurrence after surgery. Up to 20% of patients have detectable metastasis at the time of diagnosis. While the five-year survival rate of patients with OS has significantly improved with the use of chemotherapy combined with surgical treatment, attending up to 60-70%, this percentage falls to 10-30% in case of metastatic disease [2]. Lungs are the most common site of metastasis in patients with OS, followed by skeletal sites. For this reason, a thoracic CT must be performed at the time of diagnosis and in follow-up, aiming for early detection of metastases and making surgery always possible [2]. CT appearance of lung metastases is extremely heterogeneous, and differentiating a metastatic nodule from a benign lesion may be challenging. For example, the detection of calcification or ossification within a lung nodule is a typical finding of benign or inert lesions (granuloma, hamartoma, and sequelae), and these findings can also rarely occur in OS metastases. Calcification and ossification in metastases can arise through a variety of mechanisms: bone formation in tumor's osteoid origin, calcification and ossification of tumor cartilage, dystrophic calcification and ossification of tumor cartilage, dystrophic calcification, and mucoid calcification [5].

Moreover, OS lung metastases may appear a long time after initial presentation. This mechanism is known as late-relapse metastasis [4]. The majority (95%) of relapses will occur within five years of the initial diagnosis, with an average time to relapse of 1.6 years [4]. This suggests a possible mechanism of dormant metastatic cells reactivation; a large fraction of cancer cells, which have remained viable in the target organ, enters into solitary dormancy before surgical resection of the primary tumor interrupts dissemination [4]. After a variable lag time, a small minority of dormant cells undergoes reactivation and gives rise to metastatic outgrowths. Different studies suggest that dormancy and reactivation are governed by complex interactions between metastasis-initiating cells and the microenvironment of the target organ [4]. A better understanding of the clinical history of atypical metastatic features and behaviors is essential to achieve the correct diagnosis and prompt treatment.

## Conclusions

OS metastases present various types of aspects and atypical behaviors. More specifically, lung metastases may occur a long time after initial presentation, suggesting reactivation of dormant metastatic cells. This mechanism is known as late-relapse metastasis. A better understanding of these features could have a significant impact on the prognosis of patients with OS.
